# 肺癌手术患者术前焦虑或抑郁的影响因素分析

**DOI:** 10.3779/j.issn.1009-3419.2020.105.01

**Published:** 2020-07-20

**Authors:** 彦霖 杜, 永 崔, 先启 蔡, 亚莉 李, 东杰 杨

**Affiliations:** 100050 北京，首都医科大学附属北京友谊医院胸外科 Department of Thoracic Surgery, Beijing Friendship Hospital, Capital Medical University, Beijing 100050, China

**Keywords:** 焦虑, 抑郁, 肺肿瘤, Anxiety, Depression, Lung neoplasms

## Abstract

**背景与目的:**

肺癌手术患者术前焦虑、抑郁可带给患者身心双方面的危害，既往关于术前住院天数是否会使肺癌术前患者增加心理负担的研究较少。本研究旨在探讨术前住院天数对患者心理状态是否产生影响以及恶性肿瘤患者术前知情情况对其心理状态的影响。

**方法:**

以北京友谊医院胸外科处于肺癌围手术期的135例患者作为研究对象，采用一般调查问卷、Zung焦虑自评量表（self-rating anxiety scale, SAS）、抑郁自评量表（self-rating depression scale, SDS）对患者的一般情况资料、患者的焦虑/抑郁情况进行调查记录。

**结果:**

入院当天SAS得分为36.25（30.00, 42.50）分，术前1天SAS得分为37.50（31.25, 43.75）分；入院当天SDS得分为46.25（40.00, 52.50）分，术前1天SDS得分为47.50（41.25, 53.75）分。对比中国常模，入院当天焦虑患者0例，轻度抑郁患者6例，术前1天轻度焦虑患者2例，轻度抑郁患者8例；通过统计学方法分析，单一影响因素分析表明肺癌围手术期患者术前住院天数与术前焦虑、抑郁呈正相关，结果有统计学意义（*P* < 0.05）。广义线性模型分析表明其他因素如知情情况、性别、年龄、婚姻状况与术前焦虑、抑郁无明显相关性。

**结论:**

肺癌住院患者术前发生焦虑、抑郁与术前住院天数呈正相关，住院天数越多，则患者的焦虑、抑郁评分越高，医护人员应积极关注肺癌患者的心理状况，应该提倡并积极建议患者住院之前在门诊完善术前检查和术前准备，以缩短入院时间，以期降低入院后发生焦虑、抑郁等不良心理问题的风险。

## Abstract (en)

**Background and objective:**

Preoperative anxiety/depression can bring physical and mental harm to the patients with lung cancer. There is little study on whether hospital waiting time before surgery can increase the psychological burden of patients with lung cancer. The aim of this study was to investigate the preoperative anxiety and depression of patients with lung cancer in our hospital, and to analyze the related influencing factors.

**Methods:**

A total of 135 lung cancer inpatients in the Department of Thoracic Surgery of Beijing Friendship Hospital were studied. Their general information and anxiety/depression were recorded by general questionnaire, Zung self-rating anxiety scale (SAS) and self-rating depression scale (SDS).

**Results:**

The score of SAS was 36.25 (30.00, 42.50) on the day of admission, and 37.50 (31.25, 43.75) on one day before operation. The score of self-rating depression scale (SDS) was 46.25 (40.00, 52.50) on the day of admission, and 47.50 (41.25, 53.75) on one day before operation. Compared with the Chinese norm, there were 0 patient suffered from anxiety on the day of admission, and one day before operation. There were 2 patients suffered from mild anxiety; 6 patients suffered from mild depression on the day of admission, and this number went up to 8 on the day before operation. Single factor analysis showed that the hospital waiting time before surgery was positively correlated with preoperative anxiety and depression, and the results were statistically significant (*P* < 0.05). The generalized linear model analysis showed that other factors such as knowledge, gender, age and marital status had no significant correlation with preoperative anxiety and depression.

**Conclusion:**

The occurrence of preoperative anxiety in hospitalized patients with lung cancer is positively correlated to the hospital waiting time before surgery. The longer they stayed in the hospital before operation, the greater their risk of anxiety/depression. So medical staff should actively focus on the psychological condition of the patients with lung cancer, and it is strongly recommended that patients complete preoperative examination and preparation in the outpatient department, in order to reduce the waiting time before operation and reduced the risk of adverse psychological problems such as anxiety and depression.

肺癌是我国乃至全球恶性肿瘤患者死亡的主要原因之一。肿瘤患者常伴有术前焦虑、抑郁，其中以肺癌患者焦虑发生率最高，术前处于焦虑、抑郁状态会给患者带来诸多不良影响，如引起血压、心率、血糖等变化，增加镇静、麻醉类药物的用量，延长术后恢复和住院时间，甚至增加围手术期相关并发症的发生风险^[1-5]^，既往研究主要是关注肺癌患者术前的焦虑状态及其相关因素，本研究的主要目的是探讨术前住院天数增加对患者心理的不良影响，拟说明减少术前住院时间对患者的心理状态有积极影响，提示术前检查和术前准备应尽量在住院前完成。

## 1 资料与方法

### 1.1 研究对象

采用便利抽样法抽取2017年11月-2018年9月于北京友谊医院胸外科准备行手术治疗（开胸手术或者胸腔镜手术）的135例住院肺癌患者进行问卷调查。纳入标准：①经临床、影像学及病理断诊为非小细胞肺癌，且需要行开胸或胸腔镜手术患者[包括楔形切除患者和肺叶（段）切除患者]；②年龄：18岁-87岁；③病情平稳，精神正常，无交流障碍；④愿意配合调查。排除标准：①病情危重或交流障碍；②拒绝参与调查；③患者在入院后与术前知情情况发生改变；④不能完成全部调查内容或正在参加其他试验者。

### 1.2 研究方法

#### 1.2.1 调查工具

① 一般情况调查表：包括调查对象的年龄、性别、婚姻状况和对病情诊断的知情情况等；②焦虑自评量表（self-rating anxiety scale, SAS）：SAS采用4级评分，主要评定症状出现的频度。其标准为：1分表示没有或很少时间有，2分表示有时有，3分表示大部分时间有，4分表示绝大部分或全部时间都有。20个条目中有15项使用负性词陈述，按上述1-4顺序评分。其余5项（第5、9、13、17、19项）使用正性词陈述，按4-1顺序反向计分。将20个项目的各个得分相加即得粗分，用粗分乘以1.25后取整数部分得到标准分。按照中国常模结果，标准分 < 50分为无焦虑，50分-59分为轻度焦虑，60分-69分为中度焦虑，≥70分为重度焦虑^[1, 2, 6]^；③抑郁自评量表（self-rating depression scale, SDS）：SDS采用4级评分，主要评定症状出现的频度。其标准为：1分表示没有或很少时间有，2分表示有时有，3分表示大部分时间有，4分表示绝大部分或全部时间都有。20个条目中有10项使用负性词陈述，按上述1-4顺序评分。其余10项（第2、5、6、11、12、14、16、17、18、20项）使用正性词陈述，按4-1顺序反向计分。将20个项目的各个得分相加即得粗分，用粗分乘以1.25后取整数部分得到标准分。按照中国常模结果，标准分 < 53分为无抑郁，53分-62分为轻度抑郁，63分-72分为中度抑郁，≥73分为重度抑郁^[7, 8]^。

#### 1.2.2 调查方法

研究者向已纳入研究的手术患者解释研究目的，在征得患者同意的情况下，由其本人完成问卷及一般资料调查表，不能独立完成者由调查人员通过问答形式填写。首先在患者入院时即填写一般资料调查表，SDS及SAS分别让患者在入院当天及术前1天填写，所有调查问卷均为当天回收。

### 1.3 统计学方法

应用SPSS 25.0统计软件对数据进行分析。采用*Shapiro-Wilks*检验计量资料是否符合正态分布，符合正态分布的计量资料以均数±标准差表示；不符合正态分布的，以中位数和四分位数M（P25-P75）表示。入院与术前的SDS、SAS的比较采用配对*Wilcoxon*检验。采用广义线性模型对术前SDS、SAS与其他各指标进行单因素与多因素关联性研究分析。检验水准为双侧α=0.05，*P* < 0.05为差异有统计学意义。

## 2 结果

SAS入院当天得分为36.25（30.00, 42.50）分，术前1天得分为37.50（31.25, 43.75）分；SDS入院当天得分为46.25（40.00, 52.50）分，术前1天得分为47.50（41.25, 53.75）分（表 1）。其中入院当天焦虑患者0例，术前1天轻度焦虑患者2例。入院当天轻度抑郁患者6例，术前1天轻度抑郁患者8例；广义线性模型分析术前SAS、SDS评分与入院天数、知情情况、性别、年龄、婚姻状况等因素的相关性，结果为术前住院天数与术前SAS、SDS具有一定关联程度，术前住院天数越多，术前SAS、SDS评分越高，差异有统计学意义（*P* < 0.05）。其他因素如知情情况、性别、年龄、婚姻状况与术前SAS/SDS评分无明显相关性（表 2-表 4）。术前SAS、SDS评分分别与术前住院天数进行单因素关联性分析结果提示：术前住院天数与术前SDS、SAS评分具有一定关联程度，术前住院天数越多，术前SDS、SAS评分越高，差异有统计学意义（*P* < 0.05）（表 5）。

**1:** 正态性检验结果 Normality test results

Category	Numerical value (P25-P75)	*Shapiro-Wilks* test value	*P*
SDS (The day of admission)	46.25 (40.00, 52.50)	0.938	< 0.001
SDS (One day before surgery)	47.50 (41.25, 53.75)	0.976	0.019
SAS (The day of admission)	36.25 (30.00, 42.50)	0.943	< 0.001
SAS (One day before surgery)	37.50 (31.25, 43.75)	0.920	< 0.001
Age (Mean±SD, yr)	59.74±10.47	0.982	0.080
Length of stay before surgery	6.00 (2.00, 10.00)	0.848	< 0.001
SAS: self-rating anxiety scale; SDS: self-rating depression scale.			

**2:** 入院与术前的SDS、SAS比较 Comparison of SDS and SAS on the day of admission and one day before surgery

Category	The day of admission (P25-P75)	One day before surgery (P25-P75)	*z*	*P*
SDS	46.25 (42.50, 48.75)	47.50 (42.50, 48.75)	2.139	0.032
SAS	36.25 (33.75, 38.75)	37.50 (35.00, 41.25)	5.504	< 0.001

**3:** 术前SDS与各指标多因素关联性分析 Correlation analysis between preoperative SDS (one day before surgery) and various indicators

Category	*β* (95%CI)	S.E.	Wald	*P*
Length of stay before surgery	0.11 (0.03-0.19)	0.04	7.68	0.010
Informed consent status	-0.27 (-1.86-1.33)	0.81	0.11	0.740
Gender	-0.58 (-2.19-1.03)	0.82	0.50	0.480
Age	0.03 (-0.22-0.27)	0.13	0.04	0.840
Marriage	0.30 (-3.87-4.47)	2.13	0.02	0.890

**4:** 术前SAS与各指标多因素关联性分析 Correlation analysis between preoperative SAS (one day before surgery) and various indicators

Category	*β* (95%CI)	S.E.	Wald	*P*
Length of stay before surgery	0.43 (0.18-0.68)	0.13	15.72	< 0.001
Informed consent status	0.82 (-2.14-1.08)	1.08	0.41	0.520
Gender	0.83 (-3.12-0.13)	0.13	3.24	0.070
Age	0.04 (-0.05-0.11)	0.11	0.50	0.480
Marriage	2.15 (-4.36-4.07)	4.07	0.01	0.950

**5:** 术前SDS、SAS与术前住院天数的单因素关联性分析 Univariate correlation analysis of preoperative SAS (one day before surgery), SDS (one day before surgery) and length of stay before surgery

Category	*β* (95%CI)	S.E.	Wald	*P*
SDS	0.24 (0.12-0.36)	0.06	7.93	< 0.010
SAS	0.56 (0.27-0.85)	0.15	5.56	< 0.010

## 3 讨论

近年来，国内外关于肺癌住院手术患者围手术期焦虑、抑郁影响因素做了一定的研究，但术前住院天数是否会增加患者术前发生焦虑、抑郁的风险尚无定论^[9-11]^，本研究通过对本院135例肺癌手术患者的调查研究，说明了术前住院时间会增加患者术前发生焦虑、抑郁等不良心理问题的风险，因而减少术前住院时间对患者的心理状态有积极影响。本研究以我院胸外科135例肺癌住院患者为研究对象，分别在入院当天和术前1天对每一例患者进行一般情况、SDS及SAS问卷调查，经过统计学分析，患者的术前住院天数与患者术前焦虑评分和术前抑郁评分有关，术前住院天数越多，术前焦虑、抑郁评分越高，具有统计学差异（*P* < 0.05），而术前焦虑、抑郁评分与其他因素，诸如知情情况、性别、年龄、婚姻状况等无明显相关性。本研究与王伟杰团队的研究结果^[5]^一致，认为患者的焦虑、抑郁与性别、年龄无关。而冯秀琴团队^[12]^、谭晓俊团队^[13]^对肺癌手术患者的研究认为患者知晓病情后焦虑抑郁评分均明显增高，与本研究结果不一致。原因分析：上述研究中是以知情前后为时间节点，对比知情前后的焦虑、抑郁评分而得出结果，本研究中对比的是入院当天和术前1天的患者焦虑、抑郁评分，知情时间和填写调查表时间不存在相关性，以往有研究^[14-17]^表明术前会出现负性情绪过多的现象，但经历一段时间后多数患者都会表现出对疾病和手术良好的心理适应性，因此可能导致调查不一致。有研究^[18]^认为年龄是影响肺癌患者的因素，本研究纳入的患者平均年龄为59岁，最小年龄为39岁，患者以中老年人为主，其家庭负担和社会角色、心理压力等相对青少年而言无明显差异，这些因素可能会导致本研究与既往研究结果不同。

既往关于肺癌患者焦虑、抑郁的研究提出了很多影响因素，但结果并不完全一致，这可能与研究人群不同及样本量存在差异等因素有关。本研究的局限性在于样本量偏小而且研究对象集中在同一家医院，而且这可能会影响结果的推广。另外本研究在设计时主要目的是探讨两个方面对患者心理状态的影响：①术前住院天数对患者心理状态是正面影响还是负面影响；②恶性肿瘤患者术前知情对心理状态的影响。由于纳入样本量有限，因而未考虑将肺癌患者分期及合并基础疾病等情况纳入研究。我们会在今后的研究中关注这一问题，争取更全面地分析肺癌手术患者术前焦虑的影响因素。

有研究^[19, 20]^表明，相比其他恶性肿瘤患者，肺癌患者承受了更大的心理压力和病耻感，心理健康状况较差。同时新确诊肺癌患者存在焦虑和抑郁，存在抑郁的患者治疗依从性较差，平均存活时间短，仅为6.8个月。焦虑、抑郁状态是恶性肿瘤患者常见的心理反应，在很大程度上影响着患者的治疗、康复及生活质量^[21, 22]^。本研究中的结论与以往研究相比，创新之处在于通过统计学分析，证明了肺癌手术患者的术前住院天数与其发生术前焦虑、抑郁相关，这一结果可能是由于患者入院后生活环境及生活方式的改变以及缺乏家人及亲属陪伴的原因，而使肺癌住院患者更加容易发生焦虑、抑郁等不良情绪^[23-26]^。因此在临床诊疗工作中，医务人员在进行临床诊疗的同时也应积极关注肺癌患者的心理状况，提倡并积极建议患者在门诊完善术前检查、术前准备，缩短术前住院时间，以期降低入院后发生焦虑、抑郁等不良心理问题的风险，减少心理问题给患者带来的诸多不良影响，改善患者生活质量。
